# Cerebral Autoregulation in Non-Brain Injured Patients: A Systematic Review

**DOI:** 10.3389/fneur.2021.732176

**Published:** 2021-11-16

**Authors:** Yaroslava Longhitano, Francesca Iannuzzi, Giulia Bonatti, Christian Zanza, Antonio Messina, Daniel Godoy, Wojciech Dabrowski, Li Xiuyun, Marek Czosnyka, Paolo Pelosi, Rafael Badenes, Chiara Robba

**Affiliations:** ^1^Department of Anesthesiology and Critical Care, AO St. Antonio, Biagio and Cesare Arrigo, Alessandria, Italy; ^2^Department of Surgical Sciences and Integrated Diagnostics (DISC), University of Genoa, Genoa, Italy; ^3^Anesthesia and Intensive Care, Gaslini Hospital, Genova, Italy; ^4^Foundation of “Nuovo Ospedale Alba-Bra” and Department of Emergency Medicine, Anesthesia and Critical Care Division, Michele and Pietro Ferrero Hospital, Verduno, Italy; ^5^Humanitas Clinical and Research Center – IRCCS, Milan, Italy; ^6^Department of Biomedical Sciences, Humanitas University, Milan, Italy; ^7^Neurointensive Care Unit, Sanatorio Pasteur, 2 Intensive Care Unit, Hospital Carlos Malbran, Catamarca, Argentina; ^8^Anesthesia and Intensive Care, University of Lublin, Lublin, Poland; ^9^Department of Anesthesiology & Critical Care Medicine, John Hopkins University, Baltimore, MD, United States; ^10^Brain Physics Laboratory, Department of Clinical Neurosciences, University of Cambridge, Cambridge, United Kingdom; ^11^Anesthesia and Critical Care, San Martino Policlinico Hospital, IRCCS for Oncology and Neuroscience, Genoa, Italy; ^12^Department of Anesthesiology and Surgical-Trauma Intensive Care, Hospital Clinic Universitari de Valencia, Department of Surgery, University of Valencia, Valencia, Spain

**Keywords:** cerebral autoregulation, non-brain injury, neurologic outcome, sepsis, perioperative care, pediatric surgery

## Abstract

**Introduction:** Cerebral autoregulation (CA) plays a fundamental role in the maintenance of adequate cerebral blood flow (CBF). CA monitoring, through direct and indirect techniques, may guide an appropriate therapeutic approach aimed at improving CBF and reducing neurological complications; so far, the role of CA has been investigated mainly in brain-injured patients. The aim of this study is to investigate the role of CA in non-brain injured patients.

**Methods:** A systematic consultation of literature was carried out. Search terms included: “CA and sepsis,” “CA and surgery,” and “CA and non-brain injury.”

**Results:** Our research individualized 294 studies and after screening, 22 studies were analyzed in this study. Studies were divided in three groups: CA in sepsis and septic shock, CA during surgery, and CA in the pediatric population. Studies in sepsis and intraoperative setting highlighted a relationship between the incidence of sepsis-associated delirium and impaired CA. The most investigated setting in the pediatric population is cardiac surgery, but the role and measurement of CA need to be further elucidated.

**Conclusion:** In non-brain injured patients, impaired CA may result in cognitive dysfunction, neurological damage, worst outcome, and increased mortality. Monitoring CA might be a useful tool for the bedside optimization and individualization of the clinical management in this group of patients.

## Introduction

Cerebral autoregulation (CA) is a complex mechanism of brain protection against changes in cerebral perfusion pressure (CPP) in order to maintain an adequate cerebral blood flow (CBF) (1). CA works by balancing vasoconstriction and vasodilation of the cerebral vessels regulating CBF with the aim to maintain a constant CBF (1). The concept of CA was introduced approximately half a century ago and afterward it was progressively used as a parameter to help in the management of mean arterial blood pressure in relationship with CBF in the neuro-intensive care unit (neuro-ICU) settings. Indeed, monitoring CA has shown to be particularly useful in the context of acute subarachnoid hemorrhage, traumatic brain injury (TBI), and acute stroke (2–5). Although no randomized controlled trials (RCTs) assessing the effect of autoregulation on outcome are available at present, it is accepted among experts that impairment of CA can lead to secondary cerebral insult and poor outcomes also in patients without primary brain injury (6–10).

The latest guidelines from the Brain Trauma Foundation suggest to maintain a CPP of 60–70 mm Hg in patients with TBI (11); however, the concept of “one-size-fits-all” may not be appropriate, as it does not represent the physiological needs of an individual and changes of CA; more recently, experts suggest a personalized management of CA based on the intrinsic autoregulatory state and function of single patients (12). In this context, several methods aimed to individualize the assessment of CA status have been proposed (13). The gold standard for CA measurement is represented by the neuroimaging studies, which allow to obtain a direct visualization of CBF such as PET, single-photon emission CT (SPECT), and CT perfusion. However, these methods are expensive, time-consuming, and have a very limited role in the clinical context. Therefore, a number of indirect techniques aimed at CA monitoring by the bedside have been proposed and evaluated including invasive methods (based on intracranial pressure and invasive cerebral oxygenation monitoring) and non-invasive methods [based on transcranial Doppler (TCD) and near-IR spectroscopy (NIRS)]; both of them are widely adopted in the neuro-ICU population.

The aim of this study is to systematically review the role of CA in non-brain injured patients, in different clinical settings, such as surgery, sepsis, and septic shock and in the pediatric population.

## Methods

### Data Sources and Search Strategy

In this study, we adhered to the *Preferred Reporting Items for Systematic Review and Meta-Analysis Protocols* (PRISMA-P) guidelines (14). A systematic literature search was performed by using the following databases to identify relevant studies in indexed scientific journals: the PubMed, the Medical Literature Analysis and Retrieval System Online (MEDLINE) (*via* Ovid), the Excerpta Medica dataBASE (EMBASE) (*via* Ovid), and the Cochrane Central Register of Controlled Trials by using the terms: cerebral autoregulation and sepsis, cerebral autoregulation and surgery and/or perioperative, and cerebral autoregulation and non-brain injured patients with filters for humans, language (English), and time of publication (January 1, 2010 to February 28, 2021). Inclusion criteria were studies describing the measurement of CA in non-brain injured patients and its effect on outcome of patients (evaluated as mortality and/or neurological outcome). This study was limited to clinical trials, meta-analysis, RCTs, review, and systematic review. Search criteria included “CA and surgery,” “CA and sepsis and/or metabolic coma,” “CA and general intensive care,” and “CA and non-brain injured patients.” A specific literature search was then conducted for the pediatric population, with the same search strategy, but including patients <18 years. We excluded literature concerning brain injury population and not concerning CA specifically or not mentioning the effect of CA measurement on outcome. We also excluded editorials, commentaries, letters to the editor, opinion articles, reviews, meeting abstracts, and original articles lacking abstract.

### Study Selection and Data Collection

Two authors (FI and GB) selected the articles independently screening titles, abstracts, and full texts. The standardized data extraction form included aim and study design. The articles were then subdivided into three subgroups: “included” and “excluded” (if the two examiners agreed with the selection) or “uncertain” (in case of disagreement). In the case of “uncertain” classification, discrepancies were resolved by further examination performed by the two expert authors (CR and YL). We used a standardized electronic spreadsheet (Microsoft Excel, version 14.4.1; Microsoft, Redmond, Washington, USA) to extract the data from all the included studies and recording trial characteristics.

### Endpoints

The primary outcome of this study is to investigate the CA in patients without brain injury. Secondary outcome is to assess whether alteration in CA in different settings such as surgery, sepsis, and septic shock and in the pediatric population can have an effect on morbidity and mortality.

### Risk of Bias Assessment in the Included Studies

Two examiners (FI and YL) independently assessed the internal validity of the included studies and discrepancies were resolved by a third senior author (AM or CR) by using the version 2 of the Cochrane risk-of-bias tool for randomized trials (RoB 2). The RoB 2 considers five bias domains: (1) the randomization process, (2) the deviations from intended interventions, (3) missing outcome data, (4) measurement of the outcome, and (5) selection of the reported results. Finally, the overall risk of bias was calculated and, accordingly, studies were included in either high-risk/unclear risk/low-risk groups.

## Results

**Figure 1:**
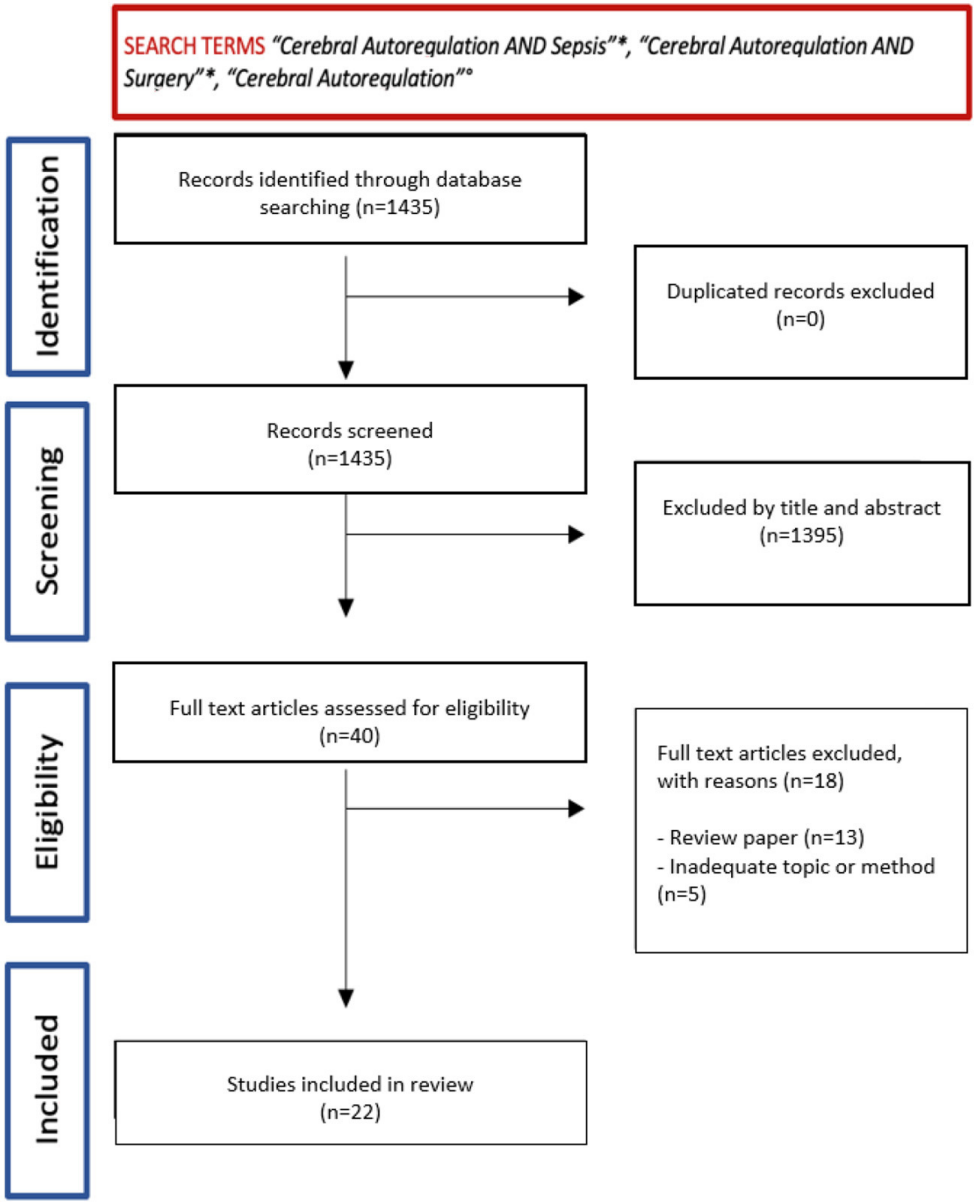
Flowchart.

After general screening, initial results from this search lead to a number of 40 studies (Figure 1, Flowchart). Among them, 22 studies were further selected, which were divided in three sections (CA in sepsis and septic shock, CA in surgery, and CA in the pediatric population) (Tables 1, 2). Specific reasons for exclusion of the studies are presented in Table 3. The risk of bias assessment reported: “low risk” for 13 papers (59%), “unclear risk” for 9 articles (41%), and none of them reported high risk of bias. The bias was mostly related to the randomization process selection of the reported results (Table 4).

**Table 1:** Summary of the included studies about CA in sepsis and pediatric population: primary findings, type of method to assess autoregulation, number of patients evaluated.

**First author**	**Year**	**Journal**	**N of patients**	**Type autoregulation**	**Design of study**	**Findings**
**CEREBRAL AUTOREGULATION IN SEPSIS AND SEPTIC SHOCK**						
Schramm et al. (15)	2012	Critical Care	30	TCD	Prospective study	AR is impaired in the great majority of patients with severe sepsis during the first two days. Impaired AR is associated with SAD, suggesting that dysfunction of CA is one of the trigger mechanisms contributing to the development of SAD.
Berg et al. (8)	2016	Scand J Clin Lab	9	TCD	Prospective study	Dynamic CA is enhanced during the very early stages of sepsis, they remain inconclusive with regard to more advanced stages of disease, because thigh-cuff deflation failed to induce sufficient MAP reductions in patients.
Bindra et al. (16)	2016	Critic Care Resusc	28	NIRS	Prospective, observational	CA is impaired early in septic shock and is independently associated with mortality at 3-month follow-up. Information based on bedside monitoring of CA in the ICU could form a valuable adjunct to guide haemodynamic optimization in patients with septic shock.
Crippa et al. (17)	2018	Crit Care	100	TCD	Prospective, observational	CA was altered in half of the patients with sepsis and was associated with the development of SABD. These findings support the concept that cerebral hypoxia could contribute to the development of SABD.
Rosenblatt et al. (18)	2020	J Intensive Care Medicine	6	NIRS / COx	Case series	In this high-fidelity group of patients with SAE, continuous, NIRS-based monitoring can identify blood pressure ranges that improve autoregulation. This is important given the association between cerebral autoregulatory function and severity of encephalopathy. Individualizing blood pressure goals using bedside autoregulation monitoring may better preserve cerebral perfusion in SAE than current practice
Berg et al. (8)	2016	Scand J Clin Lab	7	TCD	Prospective study	Cerebral CO_2_ vasoreactivity was found to be preserved in septic patients; nevertheless, and in contrast to our working hypothesis, short-term mechanical hyperventilation did not enhance dCA.
**CEREBRAL AUTOREGULATION IN PEDIATRIC CRITICALLY ILL PATIENTS**						
Easley et al. (10)	2018	Cardiol Young	57	NIRS	multicenter observational pilot study	Individual, dynamic non-invasive cerebrovascular reactivity monitoring demonstrated transient periods of impairment related to possible silent brain injury. The association between an impaired autoregulation burden and elevation in the serum brain biomarker may identify brain perfusion risk that could result in injury.
Brady et al. (19)	2010	Crit Care Medicine	54	NIRS	prospective, observational pilot study	This pilot study of COx monitoring in pediatric patients demonstrates an association between hypotension during CPB and impairment of autoregulation. The COx may be useful to identify arterial blood pressure-dependent limits of during CPB. Larger trials with neurological outcomes are indicated.
Thewissen et al. (20)	2018	Pediatr Res	22	NIRS	Prospective, observational study	Drug-related hypotension and decreased cerebral activity after intubation with low propofol doses in preterm neonates were observed, without evidence of cerebral ischemic hypoxia. CA remained intact during drug-related hypotension in 95.5% of patients. Cerebral monitoring including CA clarifies the cerebral impact of MAP fluctuations.
Joram et al. (21)	2020	Neurocritical Care	29	NIRS	Prospective observational study	CA assessment is feasible in pediatric ECMO. The first 24 h following ECMO represents the most critical period regarding CA. Impaired autoregulation is significantly more severe among patients who experience acute neurological event.

*TDC, Transcranial Doppler; AR, Autoregulation; SAD, Sepsis-associated Delirium; MAP, Mean Arterial Pressure; NIRS, Near InfraRed Spectroscopy; CVAR: Cerebrovascular Autoregulation; ICU: Intensive Care Unit; SABD, Sepsis-associated brain dysfunction; COx, cerebral oxygen index; SAE, Sepsis-associated Encephalopathy; CA, cerebral autoregulation; CPB, cardio-pulmonary by-pass*.

**Table 2:** Summary of the included studies about CA in surgery: primary findings, type of method to assess autoregulation, number of patients evaluated.

**First author**	**Year**	**Journal**	**N of patients**	**Type autoregulation**	**Design of study**	**Finding**
**CEREBRAL AUTOREGULATION DURING SURGERY**						
Caldas et al. (22)	2019	Clin Neurophysiol	67	TCD	Sistematic review	Dynamic CA was impaired after CABG surgery with CPB and was a significant independent risk factor of PD.
Ono et al. (23)	2013	Crit Care Med	410	NIRS	Prospective observational study.	Excursions of MAP below the limit of autoregulation and not absolute MAP are independently associated with for AKI. Monitoring Cox may provide a novel method for precisely guiding MAP targets during CPB.
Hori et al. (24)	2014	J Cardiothorac Vasc Anesth	110	NIRS	Prospective randomized clinical trial	The presence of delirium was not associated with perioperative blood pressure excursions, but the secondary analysis showed the association between excursion above the optimal mean arterial pressure and the severity of delirium in early PO period.
Hori et al. (25)	2014	Br J Anaesth J	491	NIRS	Prospective observational study	Excursions of MAP above the upper limit of CA during CPB are associated with risk for delirium. Optimizing MAP during CPB to remain within the CA range might reduce risk of delirium.
Hori et al. (24)	2016	Interact CardioVasc Thorac Surg	110	UT-NIRS	Prospective observational study	Excursion below optimal blood pressure during perioperative period is associated with CSA-AKI.
Sperna Weiland et al. (26)	2017	Br J Anaesth	14	TCD	Prospective study	During surgery, CA indices were similar to values determined before surgery. This indicates that CA can be quantified reliably and non-invasively using this novel method and confirms earlier evidence that CA is unaffected by sevoflurane anaesthesia.
Chuan et al. (27)	2018	Acta Anaesthesiol Scand	140	NIRS	prospective observational single centre study	In older and higher risk patients having major noncardiac surgery, impaired CA was associated with failure of cognitive recovery in the early postoperative period and with 1-month mortality and morbidity.
Goettel et al. (28)	2016	J Clin Monit Comput	133	TCD	prospective observational cohort study	The autoregulatory plateau is shortened in both young and older patients under Sevoflurane anesthesia with approximately 1 MAC. Lower and upper limits of CBF autoregulation, as well as the autoregulatory range, are not influenced by the age of anesthetized patients.
Zheng et al. (29)	2012	Neurocrit Care	9	TCD, NIRS	Prospective observational study	These results suggest that autoregulation is impaired in patients undergoing liver transplantation, even in the absence of acute, fulminant liver failure. Identification of patients at risk for neurologic complications after surgery may allow for prompt neuroprotective interventions, including directed pressure management
Nomura et al. (9)	2018	Anesthesia Analgesia	346	TCD, MRI	retrospective cohort analysis	Impaired CBF autoregulation is prevalent during CPB predisposing affected patients to brain hypoperfusion or hyperperfusion with low or high blood pressure, respectively. Small vessel, but not large vessel, cerebral vascular disease, male sex, and higher average body temperature during CPB appear to be associated with impaired CA.
Goettel et al. (28)	2017	Geriatric Anesthesia	82	TCD, NIRS	prospective observational cohort study	Impairment of intraoperative CBF autoregulation is not predictive of early POCD in elderly patients, although secondary analyses indicate that an association probably exists.
Hogue et al. (30)	2020	Semin Thorac Cardiovasc Surg	460	TCD	Prospective randomized clinical trial	Basing MAP during CPB on cerebral autoregulation monitoring did not reduce the frequency of the primary neurological outcome in high-risk patients compared with usual care but it was associated with a reduction in the frequency of delirium and better performance on tests of memory 4-6 weeks after surgery

*TDC, Transcranial Doppler; MAP, Mean Arterial Pressure; NIRS, Near InfraRed Spectroscopy; CA, Cerebral Autoregulation; ICU, Intensive Care Unit; SABD, Sepsis-associated brain dysfunction; SAE, Sepsis-associated Encephalopathy; AKI, Acute Kidney Injury; CPB, Cardiac Pulmonary Bypass; PO, postoperative; CABG, Coronary Artery Bypass Graft; PD, Post-operative Delirium; CBF, Cerebral Blood Flow*.

**Table 3:** Summary of excluded studies and reasons for exclusion.

**Excluded studies**	**Reason for exclusion**
Taccone et al., Current Vascular Pharmacology 2013	Narrative Review
Tauber et al., Expert Review of Anti-Infective Therapy 2016	Narrative Review
Donnelly et al., Critical Care 2016	Narrative Review
Goodson et al., JICM 2018	Narrative Review
Danielski et al., Molecular Neurobiology 2018	Narrative Review
Masse et al., Critical Care Medicine 2018	Not about cerebral autoregulation specifically, but about cerebral perfusion in septic patients
Gu et al., Neurotoxicity Research 2020	Narrative Review
Semenyutin et al., Frontiers in Physiology 2017	Deal with indications to surgery in patients with compromised dynamic cerebral autoregulation
Vranken et al., The Journal of Extra-Corporeal Technology 2017	Narrative Review
Lewis et al., Journal of Cardiothoracic and Vascular Anesthesia 2018	Narrative Review
Saxena et al., Presse Medicale 2018	Narrative Review
Bonow et al., Neurosurgical Focus 2019	Narrative Review
Kooi et al., Expert Review of Neurotherapeutics 2018	Narrative Review
Rhee et al., Pediatric Research 2018	Narrative Review
Jildenstål et al., Pediatric Anaesthesia 2019	Evaluate the agreement between frontal and occipital recordings of rScO_2_%
Montgomery et al., Anesthesia and Analgesia 2020	The aim is to determine the performance of the co-trending method by comparing CA metrics to data derived from TCD methods.
Kooi et al., Clinics in Perinatology 2020	Narrative Review
Govindan et al., Journal of Perinatology 2020;	Determine whether ventilator-related fluctuations in CBV

*rScO2%, regional cerebral oxygen saturation; CA, cerebral autoregulation; TDC, transcranial doppler; CBV, cerebral blood volume*.

**Table 4:** Risk of bias evaluation.

	**Randomization process**	**Deviation from intended interventions**	**Missing outcome data**	**Measurement of the outcome**	**Selection of the reported result**	**Overall**
Schramm et al. (15)	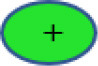	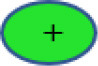	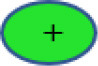	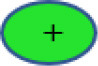	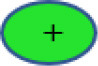	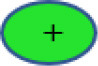
Berg et al. (8)	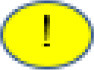	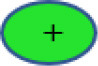	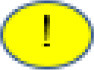	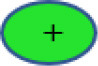	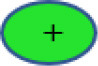	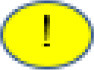
Bindra et al. (16)	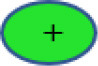	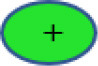	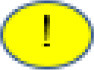	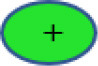	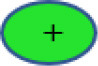	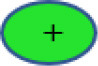
Crippa et al. (17)	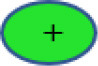	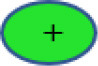	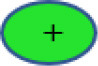	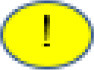	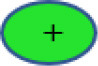	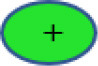
Rosenblatt et al. (18)	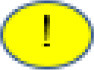	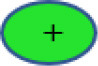	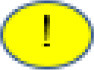	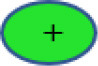	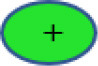	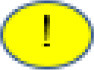
Berg et al. (8)	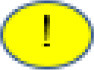	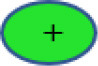	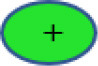	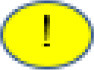	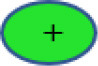	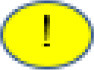
Easley et al. (10)	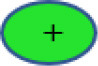	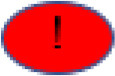	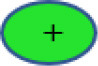	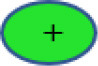	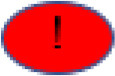	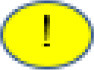
Brady et al. (19)	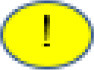	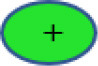	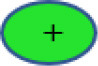	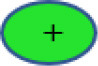	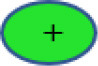	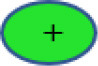
Thewissen et al. (20)	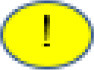	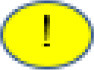	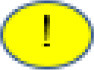	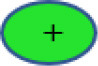	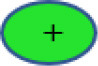	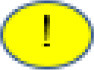
Caldas et al. (22)	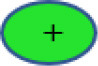	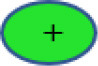	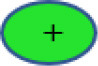	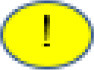	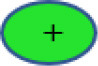	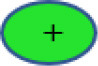
Ono et al. (23)	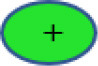	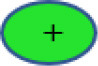	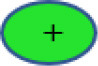	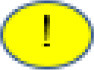	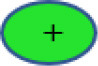	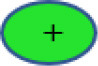
Hori et al. (24)	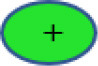	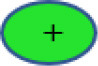	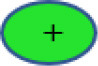	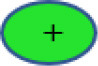	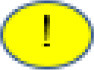	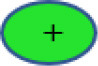
Hori et al. (25)	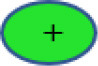	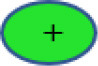	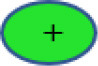	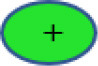	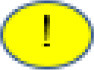	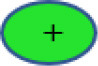
Hori et al. (24)	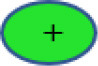	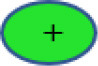	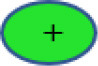	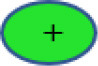	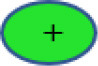	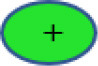
Sperna Weiland et al. (26)	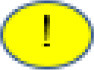	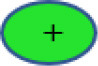	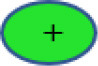	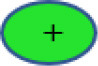	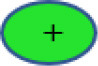	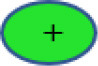
Chuan et al. (27)	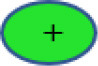	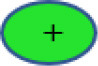	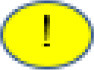	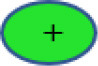	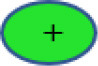	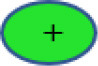
Goettel et al. (28)	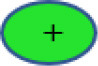	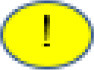	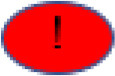	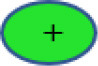	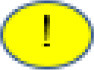	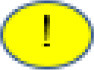
Zheng et al. (29)	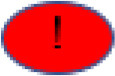	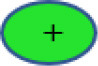	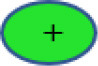	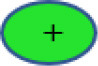	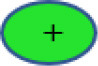	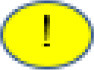
Nomura et al. (9)	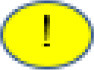	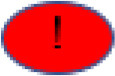	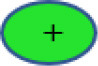	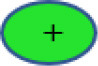	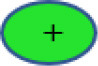	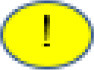
Goettel et al. (28)	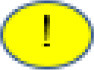	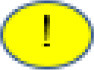	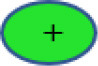	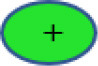	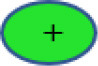	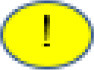
Hogue et al. (30)	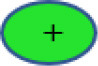	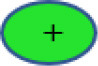	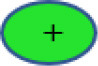	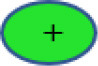	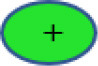	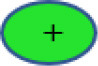
Joram et al. (21)	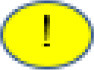	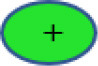	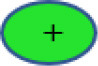	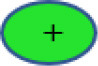	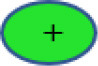	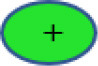

*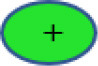 low risk of bias; 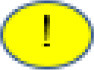 unclear risk of bias; 
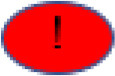
 high risk of bias*.

### Cerebral Autoregulation in Sepsis and Septic Shock

A total of six studies were screened for septic shock and sepsis (Table 1). A disruption of CA prolonged over time can lead to cerebral hypoperfusion and consequently neuronal ischemia. In a prospective, observational study, 100 adult patients with sepsis were evaluated with the hypothesis that impaired CA may lead to brain hypoperfusion and neuronal damage (17). Crippa et al. registered impaired CA in 50 patients (50%) and this represented one of the independent predictors of sepsis-associated brain dysfunction (SABD), which was diagnosed in 57 patients (57%). SABD was defined as the Glasgow Coma Scale (GCS) score <15 or when disorientation, altered thinking, or agitation was reported. In case of continuous sedation (*n* = 6), patients were considered as having SABD. The CA was evaluated by using the mean flow index (Mxa) assessed by TCD (17). The authors concluded that in SABD, brain hypoperfusion and neuronal damage were probably caused by impaired CA and this could lead to a higher morbidity and mortality in these patients. The findings of this prospective observational study support the concept that cerebral hypoxia could contribute to the development of SABD, despite its multifactorial pathophysiology. Schramm et al. also found a relationship between impaired CA and sepsis-associated delirium (SAD). Indeed, in the majority of septic patients, CA is impaired, in particular the first 2 days, suggesting an important contribution of CA in the development of SAD (15). In this study (15), 30 patients were evaluated with the average Acute Physiologic Assessment and Chronic Health Evaluation (APACHE) score of 32 ± 6. The CA was estimated by using TCD and the Confusion Assessment Method for the ICU (CAM-ICU) was assessed at day 4 when sedative medications were temporarily reduced to reach the Richmond Agitation and Sedation Scale (RASS) score greater than−2. SAD was detected in 76% of patients. This study concluded that impaired CA at day 1 was associated with the incidence of SAD at day 4 (*p* = 0.035) (15).

Cerebral autoregulation can be also evaluated to guide optimal blood pressure. Indeed, in a study including six patients (18), pharmacological sedation free with extracranial sepsis and cerebral oximetry was measured with NIRS to identify blood pressure range, demonstrating feasibility to optimize autoregulation maintaining adequate blood pressure range. In this case series, Rosenblatt et al. found an association between cerebral autoregulatory function and the development and severity of encephalopathy defined as GCS <15 but ≥ 13 (mild encephalopathy), whereas values <13 were defined as moderate or severe impairment (18). The authors concluded that individualizing blood pressure goals by using bedside autoregulation monitoring may preserve cerebral perfusion by individualizing blood pressure range in Sepsis-associated encephalopathy (SAE) (18). In another prospective observational study, Bindra et al. investigated 28 patients with early septic shock in the ICU to understand if impaired CA was associated with neurological outcome and mortality (16). This study concluded that the mortality at 3-month follow-up was independently associated with impaired CA in early septic shock. Thus, bedside monitoring of CA could adjunct important information for hemodynamic optimization of patients with septic shock. Other two studies discussed the management of CA in the early stages of septic shock (8, 31). Both were based on small groups of patients (seven and nine patients, respectively). The first study analyzed seven critically ill patients, who underwent hyperventilation (8) and the second one described healthy volunteers, who underwent lipopolysaccharide (LPS) infusion to reproduce early sepsis (31). The CA was evaluated by TCD and the authors concluded that dynamic CA (dCA) to spontaneous fluctuations in blood pressure was enhanced and cerebral carbon dioxide vasoreactivity was preserved in the early phases of sepsis, but this regulation disappeared in the late stages (8, 31).

### Cerebral Autoregulation During Surgery

A total of 12 studies were selected in the literature about CA in surgery (Table 2). A high number of articles about CA during surgery were performed in cardiac surgery, where a possible association between impaired CA and postoperative delirium (PD) was evaluated. Caldas et al. in his single-center observational and prospective study analyzed 67 patients undergoing cardiopulmonary bypass. All the enrolled subjects underwent TCD and the CAM-ICU assessment preoperatively at 24 h and 7 days after the procedure to evaluate CA and PD, respectively. Impaired CA was found in 55% of patients 24 h postoperatively and in 20% after 7 days. In addition, the authors concluded that impaired CA during coronary artery bypass graft (CABG) surgery is a significant independent risk factor for PD (*p* = 0.003) (22). Another study analyzed the use of CA with NIRS. In the prospective observational study, 110 patients who underwent cardiac surgery were analyzed (24). They were monitored with NIRS during surgery and in the first 3 h postoperatively and their CAM-ICU score was calculated on postoperative (PO) days 1 and 3. A total of 42.7% of patients presented delirium. Hori et al. observed no association between the perioperative blood pressure excursion and PD, but a secondary analysis showed higher blood pressure excursions above the optimal medial arterial pressure in the group who developed delirium postoperatively (*r* = 0.27, *p* = 0.011) (24). These data were also confirmed by a large prospective study, which analyzed 491 patients who were monitored with NIRS during surgery and the PD was assessed by the CAM-ICU. In this case, delirium was diagnosed in 9.2% of cases and the excursion of mean arterial pressure (MAP) above the upper limit of CA during cardiopulmonary bypass was independently associated with PO delirium [odds ratio (OR), 1.09; 95% CI, 1.03–1.15] (25).

In addition to a possible neuronal damage, an acute kidney injury (AKI) related to CA was investigated. Ono et al. in a large (410 patients) prospective study monitored CBF intraoperatively by using NIRS and evaluated the incidence of AKI by using the Risk, Injury, Failure, Loss of kidney function, and End-stage kidney disease (RIFLE) criteria during the first 7 days postoperatively. The results showed that MAP excursions below the lower limit of CBF autoregulation during cardiopulmonary bypass were independently associated with AKI [relative risk (RR), 1.02; 95% CI, 1.01 to 1.03; *p* < 0.0001] (23). These results were confirmed by another prospective study, which included 110 patients who underwent cardiopulmonary bypass. The CBF was monitored by NIRS during the surgery and the monitoring of AKI was performed for 2 days after surgery. A total of 27.3% of subjects developed AKI and the excursion below optimal blood pressure values during the perioperative period was associated with the development of AKI postoperatively (*p* = 0.008) (32). Other studies aimed to evaluate the principal risk factors associated to CA alteration. In a large prospective randomized clinical trial, 346 patients which underwent cardiac surgery and performed standardized general anesthesia were evaluated with preoperative TCD and cerebral MRI performed between days 3 and 5 after surgery to assess the preoperative factors, which can be associated with impaired CA in cardiac surgery. The presence of small vessel cerebral vascular disease was strongly associated with impaired CBF autoregulation (OR, 3.25; 95% CI, 1.21–8.71; *p* = 0.019). Other risk factors were male sex, high body temperature during surgery, elderly, and large vessel disease (10). Based on these evidences, it is possible to affirm that changes in CA during cardiac surgery influence clinical outcomes as rated by Caldas et al. in a recently published study (33). Also, Hogue et al. investigated the neurological impairment related to CA during cardiac surgery. A total of 460 patients were analyzed by TCD to correlate CA impairment with ischemic injury, PD, and performance on memory test 4–6 weeks after surgery. The presence of ischemic injuries was not related to intraoperative CA impairment (*p* = 0.752), instead both the delirium and memory test performance were strongly related to CA (*p* = 0.035 and *p* = 0.019, respectively) (30). However, further studies are needed to develop the therapeutic approaches in order to better understand the prognostic implications of this evidence.

In non-cardiac surgery, the role of CA was less frequently investigated and the results are discordant. Two large studies are available in scientific literature. The first one (27) is prospective and it is focused on PD and CA in major non-cardiac surgery. A total of 110 patients were included, who were intraoperatively monitored by NIRS and PO cognitive disorders were recorded. Chuan et al. concluded that impaired CA in these patients was associated with failure of cognitive recovery in the early PO period (*p* = 0.02) with 1-month mortality and morbidity (*p* = 0.04) (27). The second one (28) also investigated an association between impaired CA and PO cognitive dysfunction (POCD). Indeed, Goettel et al. recruited 86 patients who underwent non-cardiac surgery and standardized anesthesia and were intraoperatively evaluated with both the TCD and NIRS. In addition, C-reactive protein (CRP) and two biomarkers of neuronal injury neuron-specific enolase (NSE) and S100β protein were dosed on PO days 2 and 7. The study participants completed a battery of neuropsychological tests at baseline and 1 week after surgery and 3 months postoperatively. Authors concluded that dysregulation of CBF was not predictive of cognitive dysfunction after surgery, but there may be an association not yet fully explained (28). The risk factors associated with impaired CA in non-cardiac surgery were also evaluated in a study including 133 patients who underwent sevoflurane anesthesia during non-cardiac major surgery and subsequently they were divided in two groups depending on age. CBF thresholds were not influenced by the age of patients (*p* = 0.075) and autoregulatory plateau was shortened in both the groups during anesthesia with sevoflurane (34). In contrast, Sperna et al. evaluated CA in general anesthesia with TCD before surgery during 3 min of paced breathing at 6, 10, and 15 beats per min (bpm) and during surgery. The authors concluded that sevoflurane did not affect CA because preoperative and intraoperative values were superimposable (26). Finally, one study investigated the role of CA in liver transplant surgery. In this study, six patients were retrospectively analyzed with TCD to evaluate CA and its changes in perioperative period. Authors revealed marked alterations of CBF and they concluded that direct pressure management and others neuroprotective measures may reduce the risk for neurological complications in this group of patients (29).

### Cerebral Autoregulation in the Pediatric Critically Ill Population

With respect to CA in the pediatric population, four studies were included (Table 1). Even in the pediatric population, the most studied field for CA use is the cardiac surgery. In the first study included (19), the lower limit of autoregulation during cardiac surgery was measured; hypotensive events were associated with increased values of the cerebral oximetry index (Cox) (*p* < 0.0001) and the mean lower limits of pressure autoregulation were 42 ± 7 mm Hg. Authors concluded that despite larger studies must confirm these findings, COx can be useful to determine the arterial blood pressure-dependent limits of autoregulation in the pediatric population (19). Another study (10) conducted in cardiac surgery aimed to detect brain hypoperfusion injuries by elevation in serum glial fibrillary acidic protein levels due to impaired cerebrovascular reactivity. A total of 57 children were analyzed and transient period of compromised CA was detected with dynamic noninvasive cerebrovascular reactivity monitoring that showed transient periods of impairment related to possible silent brain injury (10). Out of cardiac surgery settings, CA after propofol infusion in the pediatric population was investigated. During general anesthesia, hypotension due to low dose of propofol infusion before endotracheal intubation did not lead to cerebral hypoxia and with stable levels of regional cerebral oxygen saturation. Unharmed CA drug-related hypotension was detected, underlining its importance during MAP fluctuations (20).

Another study evaluated CA during Extracorporeal Membrane Oxygenation (ECMO) treatment. This prospective observational study analyzed 29 children, who required ECMO support; in particular, the CA was evaluated with NIRS (21). In this study, 34.5% experienced acute neurological events; it concluded that the first 24 h following ECMO is the most critical period regarding CA and altered CA is associated with acute neurological events (*p* = 0.04) (21).

## Discussion

In this study, we set out to describe how CA can be modified in patients without brain injury in sepsis/septic shock during surgery and in pediatric critically ill patients. Impaired autoregulation in these pathological situations can result in cognitive dysfunction, neurological damage, increased morbidity, and mortality even in non-brain injured patient (6–9). These pathological situations can alter the autoregulatory mechanisms and values, thus modifying the ability of CA to preserve good CBF and prevent possible secondary neuronal injury (8, 17, 24).

In sepsis and septic shock, the systemic alterations that involve compromise in CA have been studied by several authors for whom the condition of SABD has also been defined (24). Despite this, the timing at which impaired CA begins with respect to the onset of the disease still needs further investigations (33). Furthermore, a correlation was found between impaired CA and outcome and mortality of septic patients independently from baseline septic disease severity (16, 18). The lack of homogeneous outcomes found in the studies included can be explained by the different settings applied and makes difficult to draw any conclusion regarding the possible effects of brain monitoring on outcome. The presence of sedation during neurological evaluation, the use of different scores for neurological impairment, and CA evaluation methods can also impact on the reproducibility of the results; nevertheless, all the studies suggest that measuring CA is safe and feasible and can potentially provide important information regarding intracerebral changes during the ICU stay and possibly improve outcomes (16–18). The analyzed studies confirm the presence of frequently altered CA in sepsis or septic shock, mostly during latest phases of sepsis and altered CA is the important risk factor for cognitive dysfunction (15, 17).

Important consequences of impaired CA were also found in the PO period. The studies included were mainly conducted in the context of cardiac surgery, in particular during cardiopulmonary bypass in which the cause of PD must be sought together with other risk factors (22, 24, 25). In support of this theory, an increased level of neuronal biomarkers was found in the serum of patients with impaired CA in the PO period, but further studies are needed to fully understand the association between POCD and altered brain flow as a consequence of the loss of CA (28). Besides cardiac surgery, in a study conducted in liver transplant surgery, we found that blood pressure management and neuroprotective measures may help in reducing the neurological complications (29). In this pathological setting, the alteration of CA is related to PO neurological and cognitive impairment (22, 27, 34), but it is not the only one comorbidity related to altered CBF. Indeed, also AKI was associated with altered CA (23, 32).

In the pediatric population, CA was evaluated mainly during cardiopulmonary bypass surgery (10, 19). An increase in PO serum neurological biomarkers following impaired CA was also detected in pediatric patients. This has been related to major fluctuations in MAP and intraoperative hypotension (10). Although evidence is limited in this setting, even in this population, the use of CA monitoring seems to be beneficial to early detect the intracerebral complications (10).

The CBF was evaluated mostly by NIRS and TCD. In case of TCD, the correlation between systemic MAP and mean flow velocity (FVm) in the middle cerebral artery was used to evaluate CA (Mx index) (15, 22, 33). Instead, in case of NIRS, the CA can be monitored by the Pearson's correlation coefficient between MAP and NIRS signals to generate the variable COx. When MAP is within the limits of CBF autoregulation, Mx and Cox approach zero or are negative, but when MAP is outside the limits of autoregulation, Mx and Cox have positive values, indicating that CBF is blood pressure passive (16, 25). Sperna et al. evaluated CA with an innovative method by using paced breathing. Indeed, the blood pressure and CBF velocity fluctuate spontaneously around the two predominant frequencies. The low frequency is due to baroreflex-mediated sympathetic nervous system activity and the high frequency is due to respiration. By using “frequency domain” analysis, the power of these fluctuations can determine the efficacy of CA (26).

This study has some limitations. First, this study summarizes the available evidence. There are still inadequate data describing the impact of CA on outcome and its clinical use in both the adult and pediatric population without brain injury. Second, the studies described in our review included a small number of patients and are extremely heterogeneous with often important methodological limitations. This makes difficult to draw any conclusion and makes a meta-analysis of the available evidence unviable.

## Conclusion

Impaired autoregulation in different pathological conditions can result in cognitive dysfunction, neurological damage, worse outcome, and increased mortality, even in non-brain injured patients. The analyzed studies show an association between alteration in CA and outcome; however, the heterogeneity of the studies and the low level of quality in the study design and methods further suggest that more in-depth investigations are needed, especially considering the different subgroups.

## Data Availability Statement

The raw data supporting the conclusions of this article will be made available by the authors, without undue reservation.

## Author Contributions

FI, YL, GB, and CZ contributed to the analysis of scientific literature and writing of manuscript. AM, DG, WD, LX, MC, PP, and CR contributed to the revision of manuscript and fundamental conceptual contribution. RB reviewed the manuscript and added fundamental contribution. All authors contributed to the article and approved the submitted version.

## Conflict of Interest

The authors declare that the research was conducted in the absence of any commercial or financial relationships that could be construed as a potential conflict of interest.

## Publisher's Note

All claims expressed in this article are solely those of the authors and do not necessarily represent those of their affiliated organizations, or those of the publisher, the editors and the reviewers. Any product that may be evaluated in this article, or claim that may be made by its manufacturer, is not guaranteed or endorsed by the publisher.
