# Supplementary material for: Cerebral Autoregulation in Non-Brain Injured Patients: A Systematic Review
Source: Front Neurol. 2021 Nov 16;12:732176. doi: 10.3389/fneur.2021.732176 (PMC8660115; doi:10.3389/fneur.2021.732176)
Supplement: Supplementary file 1 [file Data_Sheet_1.docx]

SUPPLEMENT MATERIAL

An article [1] was excluded because it dealt with the strength of method of CA monitoring, but not in relation to its specific changes during surgery. Semenuytin et al 2017 was excluded because CA was not assessed as a parameter to evaluate neurological implication and its fluctuations in surgery [2], but as a surgery indication for carotid endoarterectomy (CEA) in patients with severe internal carotid artery stenosis. In the group of “cerebral autoregulation in pediatrics population” two articles were excluded: in particular, in the first study [3], the aim was to find the agreement between two different methods of monitoring CA and not its effect on outcome, and in the other case [4] because it dealt with fluctuations in cerebral blood volume during mechanical ventilation and not in CA changes [3,4].

Bibliography

# *Montgomery D, Brown C, Hogue CW, Brady K, Nakano M, Nomura Y, Antunes A, Addison PS. Real-Time Intraoperative Determination and Reporting of Cerebral Autoregulation State Using Near-Infrared Spectroscopy. Anesth Analg. 2020 Nov;131(5):1520-1528. doi: 10.1213/ANE.0000000000004614. PMID: 33079875; PMCID: PMC7319873.*

# *Semenyutin VB, Asaturyan GA, Nikiforova AA, Aliev VA, Panuntsev GK, Iblyaminov VB, Savello AV, Patzak A. Predictive Value of Dynamic Cerebral Autoregulation Assessment in Surgical Management of Patients with High-Grade Carotid Artery Stenosis. Front Physiol. 2017 Nov 2;8:872. doi: 10.3389/fphys.2017.00872. PMID: 29163214; PMCID: PMC5673646.*

# *Govindan V, Govindan R, Massaro AN, Al-Shargabi T, Andescavage NN, Vezina G, Murnick J, Wang Y, Metzler M, Cristante C, Swisher C, Reich D, Plessis AD. Cerebral venous volume changes and pressure autoregulation in critically ill infants. J Perinatol. 2020 May;40(5):806-811. doi: 10.1038/s41372-020-0626-0. Epub 2020 Mar 10. PMID: 32157219; PMCID: PMC7224055.*

# *Jildenstål P, Sandin J, WarrènStomberg M, Pålsson J, Ricksten SE, Snygg J. Agreement between frontal and occipital regional cerebral oxygen saturation in infants during surgery and general anesthesia an observational study. Paediatr Anaesth. 2019 Nov;29(11):1122-1127. doi: 10.1111/pan.13743. Epub 2019 Oct 8. PMID: 31536668.*
